# Supplementary material for: Opicapone Improves Global Non-Motor Symptoms Burden in Parkinson’s Disease: An Open-Label Prospective Study
Source: Brain Sci. 2022 Mar 12;12(3):383. doi: 10.3390/brainsci12030383 (PMC8945982; doi:10.3390/brainsci12030383)
Supplement: Supplementary file 1 [file brainsci-12-00383-s001.zip › brainsci-1621589-supplementary.pdf]

Table S1. Change in the score of the NMSS and its domains between the visits of the study: V0 (N=33), V1 (N=31), V2 (N=30).

	V0	V1	V2	p^a	p^b	p^c
**NMSS total score**	71.67 ± 37.12	57.13 ± 34.92	52.1 ± 34.76	**0.002**	**0.001**	0.202
- Cardiovascular	5.3 ± 9.21	6.04 ± 12.11	4.03 ± 6.03	0.346	0.699	0.483
- Sleep / fatigue	33.08 ± 19.02	21.63 ± 18.5	19.82 ± 16.4	**<0.0001**	**0.001**	0.999
- Mood / apathy	22.22 ± 22.58	14.96 ± 17.97	11.87 ± 14.82	**0.001**	**0.001**	0.223
- Perceptual symptoms	1.59 ± 4.76	2.06 ± 6.12	1.88 ± 4.5	0.334	0.588	0.574
- Attention / memory	13.55 ± 15.8	11.82 ± 16.65	8.6 ± 19.62	0.091	0.216	0.070
- Gastrointestinal symptoms	19.44 ± 16.27	19.53 ± 16.33	15.41 ± 16.36	**0.029**	0.177	0165
- Urinary symptoms	32.57 ± 26.01	25.89 ± 18.16	34.49 ± 26.02	0.726	0.064	0.033
- Sexual dysfunction	15.78 ± 26.2	21.77 ± 26.67	22.98 ± 30.97	0.099	0.196	0.977
- Miscellaneous	21.96 ± 18.56	16.53 ± 15.05	12.09 ± 14.11	**0.021**	0.110	0.154

P values were computed using the Wilcoxon signed-rank test. The results represent mean ± SD. Domains of the NMSS were expressed as a percentage to be able to establish comparisons on their severity between them; p^a, V2 vs V0; p^b, V1 vs V0; p^c, V2 vs V1.

NMSS, Non-Motor Symptoms Scale.
